# Effect of Mini-invasive Floating Metatarsal Osteotomy on Plantar Pressure in Patients With Diabetic Plantar Metatarsal Head Ulcers

**DOI:** 10.1177/1071100720976099

**Published:** 2020-12-17

**Authors:** Eran Tamir, Michael Tamar, Moshe Ayalon, Shlomit Koren, Noam Shohat, Aharon S. Finestone

**Affiliations:** 1 Department of Orthopaedic Surgery, Shamir Medical Center, Zerrifin, Israel; 2 Maccabi Health Services, Tel Aviv, Israel; 3 The Faculty of Medicine, Tel Aviv University, Tel Aviv, Israel; 4 The Academic College at the Wingate Institute, Netanya, Israel; 5 Diabetes Unit, Shamir Medical Center, Zerrifin, Israel

**Keywords:** diabetic foot ulcer, metatarsal osteotomy, minimally invasive surgery, peak planter pressure

## Abstract

**Background:**

Distal metatarsal osteotomy has been used to alleviate plantar pressure caused by anatomic deformities. This study’s purpose was to examine the effect of minimally invasive floating metatarsal osteotomy on plantar pressure in patients with diabetic metatarsal head ulcers.

**Methods:**

We performed a retrospective case series of prospectively collected data on 32 patients with diabetes complicated by plantar metatarsal head ulcers without ischemia. Peak plantar pressure and pressure time integrals were examined using the Tekscan MatScan prior to surgery and 6 months following minimally invasive floating metatarsal osteotomy. Patients were followed for complications for at least 1 year.

**Results:**

Peak plantar pressure at the level of the osteotomized metatarsal head decreased from 338.1 to 225.4 kPa (*P* < .0001). The pressure time integral decreased from 82.4 to 65.0 kPa·s (*P* < .0001). All ulcers healed within a mean of 3.7 ± 4.2 weeks. There was 1 recurrence (under a hypertrophic callus of the osteotomy) during a median follow-up of 18.3 months (range, 12.2-27). Following surgery, adjacent sites showed increased plantar pressure and 4 patients developed transfer lesions (under an adjacent metatarsal head); all were managed successfully. There was 1 serious adverse event related to surgery (operative site infection) that resolved with antibiotics.

**Conclusion:**

This study showed that the minimally invasive floating metatarsal osteotomy successfully reduced local plantar pressure and that the method was safe and effective, both in treatment and prevention of recurrence.

**Level of Evidence:**

Level III, retrospective case series of prospectively collected data.

Foot ulcers are common among patients with diabetes, with a reported annual incidence of 2% to 6%.^1,7,23,40^ More than half of these ulcers become infected, and approximately 15% to 20% of the infected ulcers lead to some level of amputation.^22,36^ The 2 major factors that contribute to the development of these ulcers are neuropathy due to a lack of protective sensation and foot deformity that results in increased pressure below the affected metatarsal head. This leads to hypertrophic callus formation and increased risk for local ulcers.^4^ Consequently, plantar ulcers at the level of the metatarsal heads are common and represent 22% of all foot ulcers.^28^

In the absence of ischemia or infection, this type of ulcer usually heals promptly when offloaded.^26^ Total contact casting is still the most efficient offloading method of treating these ulcers,^18,21,26^ but it does not change the underlying mechanism causing the pathology. To prevent recurrence, offloading of the affected areas must continue for a long period with special shoes and orthotics.^10,11,29,35^ Although considered a first line of therapy, conservative treatment entails a considerably high recurrence rate with 40% recurrence at 1 year and 60% recurrence at 3 years. Even after successful resolution of a plantar ulcer, it is not considered “cured” but is more appropriately termed as “in remission.”^3,4,13^

Operative offloading is indicated when conservative treatment has failed to heal the ulcer or prevent its recurrence. In contrast to conservative treatment, surgery is aimed to prevent recurrence by correcting the underlying foot deformity.^6,30-32^ Recently, a minimally invasive operative procedure has been described involving a distal metatarsal osteotomy for the treatment of plantar metatarsal head ulcer.^6,16,30,34^ Although the distal metatarsal osteotomy has been hypothesized to address plantar ulcers by redistributing plantar pressures along the forefoot, there has not yet been any literature reporting clinical pedobarographic data following distal metatarsal osteotomy. Based on clinical experience, we hypothesized that the pressure would decrease under the head of the osteotomized metatarsal and increase under adjacent metatarsal heads.

The purpose of this study was to assess the effect of minimally invasive floating distal metatarsal osteotomy on plantar pressures in a cohort of patients and examine whether the clinical improvement is associated with reduction in plantar pressure.

## Methods

### Study design and patients

A retrospective case series of prospectively collected data on patients with diabetic foot ulcers treated by minimally invasive floating distal metatarsal osteotomy at the Maccabi HaShalom outpatient medical center in Tel Aviv, Israel . The IRB approved the study (Assuta Medical Center permit 0093-17-ASMC) and all patients signed informed consent. Study inclusion criteria were as follows: (1) had known diabetic neuropathy (positive, ≤5/8, 5.07/10 g monofilament sensory test)^27^; (2) a single neuropathic plantar ulcer at the level of metatarsal heads 2-5; and (3) were scheduled for minimally invasive floating distal osteotomy or had performed surgery up to 15 months prior to recruitment and had preoperative plantar pressure measurements. Study exclusion criteria were as follows: (1) lack of palpable pedal pulses or ankle brachial pressure index less than 0.7; (2) age <18 years or unable to sign informed consent; (3) unable to walk steadily with no support for 50 m; (4) an active infection of the foot or suspicion of osteomyelitis; and (5) weight > 120 kg, or body mass index >40, because of doubts regarding the accuracy of the pressure plate in these patients. Recruiting was started on May 1, 2018, with the intention of recruiting 44 subjects within 1 year. Because of technical restrictions, we stopped recruiting on January 31, 2019, after the 43rd case report form was issued (even though eventually only 32 subjects were included in the pedobarographic analysis, as detailed in Table 1 and the legend).

**Table 1.** Adverse Events and Complications.

OBS^a^	Surgery		Complications and Response				
	Side	MT	Months	Description	SAE at 1 y	Related	Comment
4	Rt	3	6	Severe infection, hospitalization	Yes	No	Other foot
5	Lt	3	7	Rt heel ulcer		No	Other foot
			12	Rt tip of toe ulcers, 2,3		No	Other foot
			16	Rt 5 PIP OM		No	Other foot
				Asymptomatic nonunion^b^			
6	Rt	4	9	Lt fifth MT ulcer		No	Other foot
			20	Lt fourth MT ulcer		No	Other foot
9	Rt	5	6	Recurrence (osteotomy site)		Yes	Reoperated: more proximal osteotomy
12	Lt	4,5	11	Lt tip of second toe ulcer		No	
			11	Lt tip of fourth toe ulcer		No	
13	Lt	2	18	Lt fourth MT ulcer		No	18 mo
14	Lt	5	11	Lt tip of second toe ulcer		No	
15	Lt	2,4	12	Rt third MT ulcer		No	Other foot
23	Rt	2	13	Rt third MT ulcer (grade A0)		No	13 mo
24	Lt	5	5	Foot burn		No	
			11	Transfer lesion^c^ to Lt fourth MT		Yes	Reoperated
27	Lt	5	0	Severe infection, hospitalization	Yes	Yes	
29	Rt	3	0	Infection, oral antibiotics		Yes	
31	Rt	4	14	Rt hallux amputation		No	14 mo
32	Lt	4	6	Transfer lesion^c^ to Lt fifth MT		Yes	Reoperated
33	Rt	2		Asymptomatic nonunion^b^			
34	Lt	3	12	Rt first MT OM, head resected		No	Other foot
				Asymptomatic nonunion^b^			
37	Rt	4	6	Lt fifth MT ulcer		No	Other foot
42	Lt	5	4	Transfer lesion^c^ to Lt first MT		Yes	Not operated
43	Rt	4		Asymptomatic nonunion^b^			
			11	Transfer lesion to Rt first MT		Yes	Not operated

Abbreviations: Lt, left; MT, metatarsal; OM, osteomyelitis; PIP, proximal interphalangeal joint; Rt, right; SAE, serious adverse event.

a Observation count in the original allocation (there were 7 screening failures excluded, 2 numbers not allocated and 2 dropouts).

b Asymptomatic nonunions are reported for completeness even though they were not considered complications.

c A transfer lesion is a metatarsal ulcer that developed adjacent to the index ulcer during the first 12 months following the procedure.

Demographic and clinical data collected included age, gender, years of diabetes, type of antihyperglycemic medications, weight, creatinine, and glycated hemoglobin (HbA_1C_) levels. Ulcer parameters documented included duration, location, size, and grade (Texas classification^20^), time to operative wound healing, time to ulcer healing, and postoperative complications. Ulcer area was approximated as the product of the length, width, and π/4. The operative method and postoperative management was similar to that described previously.^30^ Full weightbearing in a postoperative shoe was permitted immediately, and patients returned to normal shoes after 2 weeks.^30^ Patients were followed weekly until operative wound closure and then as clinically necessary. They were recalled for mandatory 6- and 12-month follow-up, and their HMO medical files were reviewed periodically to verify their status and that any relevant medical conditions treated by other clinicians were also documented (as patients rarely change their HMO in our country, this provides almost 100% follow-up).

Clinical outcomes assessed included time to operative wound healing, time to ulcer healing, and postoperative complications. Potential complications included operative site infection, local recurrence, transfer ulcers, and symptomatic nonunion. All adverse events are reported (Table 1). Serious adverse events (SAEs) were defined according to the FDA ICH (Food and Drug Administration, International Council for Harmonization) criteria (life threatening, death, hospitalization/prolongation of hospitalization, persistent or significant disability/incapacity, or that an intervention was required to prevent such an event). Transfer lesions were not designated as SAEs if they did not lead to hospital admission or infection, even if they did lead to a repeat mini-invasive outpatient procedure. A recurrent ulcer was defined as an ulcer that developed below the same metatarsal head that caused the primary ulcer and was osteotomized. A transfer lesion was defined as a metatarsal ulcer that developed adjacent to the index ulcer during the first 12 months following the procedure.

### Pedobarographic Measurements

All patients had pedobarographic measurement just before surgery and 6 months after the surgery. Dynamic plantar pressures were recorded during level barefoot walking at the patient’s natural cadence using the Tekscan MatScan system (Boston, MA). The system consists of a 5-mm-thick floor mat (432 × 368 mm), with 2288 resistive sensors (1.4 sensors/cm^2^), a sampling frequency of 50 Hertz (Hz) and has been shown to have moderate to good reliability,^39^ as has the 2-step gait initiation protocol in diabetic neuropathic feet.^8,9^ The software also calculates the time the foot was in contact with the plate (stance phase). A possible explanation for changes in pedobarographic measurements is a change in walking pattern. This is commonly assessed by measuring ground contact time. Pooled preoperative and postoperative contact times were 900 and 867 milliseconds, respectively, representing an insignificant difference of less than 4%.^33^

Subjects were weighed and the mat software was calibrated by their standing on the mat for a few seconds. Each subject was trained to walk barefoot at his natural pace while stepping on the floor mat with the foot placed within the area where the sensors are located and to continue walking for several steps. Four to 5 test trials per protocol were conducted for the subject to familiarize with the protocol and to determine the starting position from the mat for successful execution. The measurement was performed 10 times, measuring the reference foot only. If the measurement appeared suboptimal, either because of inadequate foot placement on the floor mat or unusual pedobarographic results, the measurement was repeated until 10 good measurements were achieved.

Peak pressure (kPa), representing the maximum pressure recorded under the specific foot zone and the pressure time integral (kPa·s), representing the overall pressure that has been applied over time were analyzed. Masking (selecting a 2×2 sensors square) was performed manually to examine the area under each of the metatarsal heads (and under the hallux and the heel for controls). The Tekscan software (composite-based calculation) calculated the maximum pressure over the sensors in the mask for every time frame, and then presented the highest of these maximum pressures of all the time frames (peak pressure). The pressure time integral summates the product of the pressures in each time frame in the stride and the duration of the time frame.

### Data Analysis / Statistics

Clinical data on subjects were presented as means ± SD for normally distributed variables and median with interquartile range (IQR) where the distribution was not normal (eg, age and durations). Means of plantar pressures and pressure time integrals for the 10 times performed were calculated and the delta between preoperative and postoperative was calculated using paired *t* test. Further analysis included repeated measures residual maximum likelihood estimation using PROC MIXED to assess the effect of the osteotomy, the location of the osteotomy and the individual. To examine the relative effect of surgery on the pressures under each metatarsal head, we grouped metatarsals as “osteotomized,” “adjacent,” and “far.” The change in mean peak pressure was then calculated for each metatarsal group (osteotomized, adjacent, and far) and classified as either increased, decreased, or having no change. No change was defined as having less than 5% change.

Predefined sample size calculation based on finding a decrease from a maximum sensor pressure of 180±65 to 157±49, α = 0.05, β = 0.2, SD of difference = 60; single-sided analysis indicated we needed 44 subjects (pairs of observations).^14^ Missing data in 2 patients due to their withdrawal of participation in follow-up pedobarographic measurements was managed by their removal from the main analysis. They consented to telephone clinical follow-up and digital medical file review, so data on complications are complete. Reporting was performed according to the STROBE statement checklist.

## Results

Overall, 34 patients met the inclusion criteria and were recruited to the study. Two of these patients declined coming for follow-up plantar pressure measurements and were therefore excluded from the final analysis. Both dropout patients were followed up by phone and had no complications during the follow-up period. The mean age of the 34 patients that were included in the study was 60.1 ± 7.5 years. All but 3 were males. All patients had type 2 diabetes, for a mean of 19.1 ± 10.1 years. Current treatment for diabetes included 2 patients on diet alone, 9 on noninsulin antihyperglycemic medications, and 22 on insulin or a combination of insulin and other antihyperglycemic agents. The subject’s mean weight was 90.1 ± 12.2 kg, body mass index 30.2 ± 4.0. Their HbA_1c_ was 7.9% ± 1.7% (63 ± 18 mmol/mol), and their mean creatinine was 1.5 ± 1.1 mg/dL. The ulcers were reported as having been present for a median of 1.5 months (range 0.5-18, IQR 1-5). The grades were Texas classification A0 in 3, A1 in 30, and A2 in 1 subject and their mean area was 97.9 ± 86.6 mm^2^ (range 19.6-392.7 mm^2^, median 78.5, IQR 27.5-94.2 mm^2^).

Of the 34 subjects operated on (17 right side and 17 left side), the primary metatarsal was the second in 8 subjects, third in 7, fourth in 6, and fifth in 13. Five of the subjects were operated on at the same session on the fourth metatarsal in addition to the primary metatarsal (3 subjects on the fourth in addition to the third, 1 subject on the fourth in addition to the second, and 1 subject on the fourth in addition to the fifth).

The mean clinical follow-up was 18.6 months (median: 18.4, range 12.2-27.5). The operative wound had healed in 32/34 (94%) patients at the first visit. The ulcer resolved in all 31 patients with ulcers (100%, Texas grades A1 and A2) within a mean of 3.7 weeks (SD 4.2, median 3, range 1-23, IQR 2-4).

There were 2 cases of operative site infections that recovered with antibiotics: one was admitted to hospital and received parenteral antibiotics (therefore designated an SAE) and the other was successfully treated with oral antibiotics. There were 4 cases of transfer lesions under the heads of adjacent metatarsals (12.5%) and 1 recurrence under the callus formed at the osteotomy site (3.0%). Of these, 2 were treated conservatively, and 3 had further off-loading surgery that was successful. Of the 39 osteotomies, 4 (10.3%) resulted in asymptomatic nonunion (that did not bother the patients nor have any negative sequela). Overall, 23 adverse events/complications occurred in 18 patients during the entire follow-up period; 16 of these events were not related to surgery (Table 1). In total, 2 patients had SAEs: 1 deep postoperative wound infection and 1 foot infection not related to the surgery.

**Figure 1.**
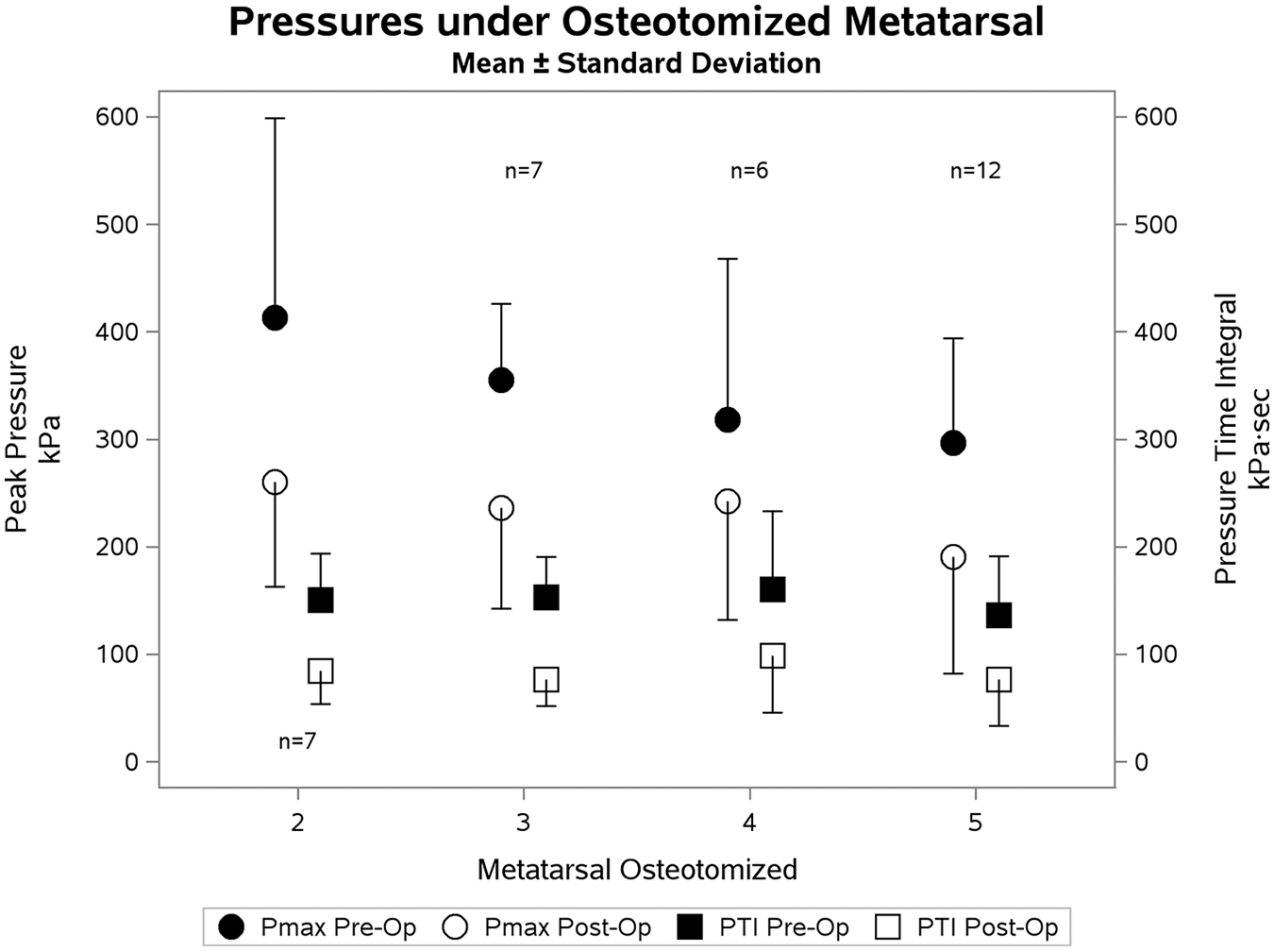
Changes in peak pressures (Pmax) and pressure time integrals (PTIs) under metatarsal heads from preoperation to 6 months postoperation. Units: peak pressures on left-hand scale, kilopascal; pressure time integrals on the right-hand scale kilopascal·seconds. Statistics: paired *t* test.

**Figure 2.**
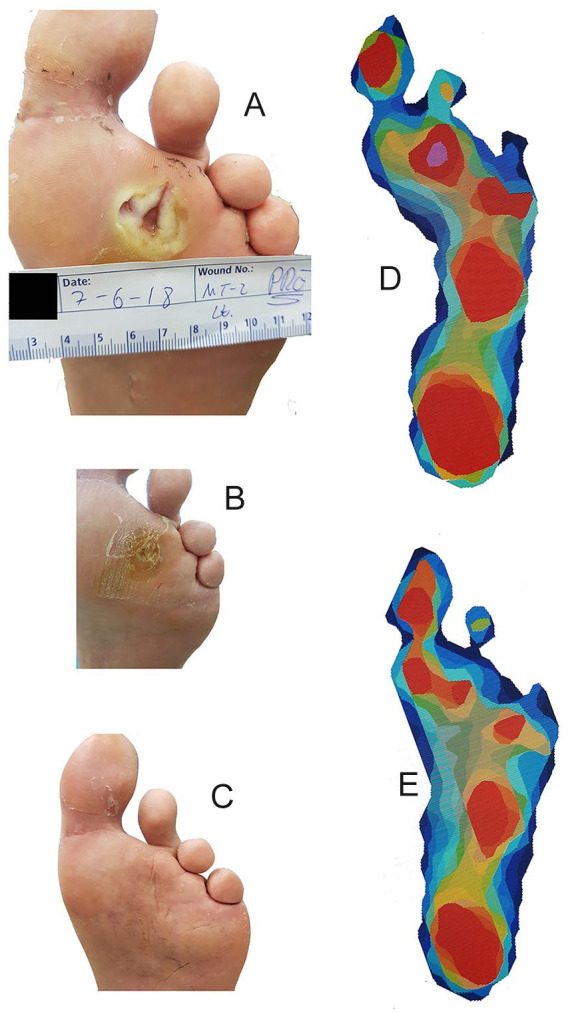
Clinical photographs and pedobarographs presenting plantar peak pressures for patient 13. (A) Preoperation; (B) 2 weeks postoperation; (C) 6 months postoperation; (D) preoperation; (E) 6 months postoperation. Pedobarographs are inverted for easy comparison with clinical photographs. The peak pressure under the second metatarsal decreased from 474±45 to 239±27 kPa (*P* < .0001, *t* test), and the peak pressure under the second metatarsal increased from 210±44 to 311±29 kPa (*P* < .0001, *t* test).

**Figure 3.**
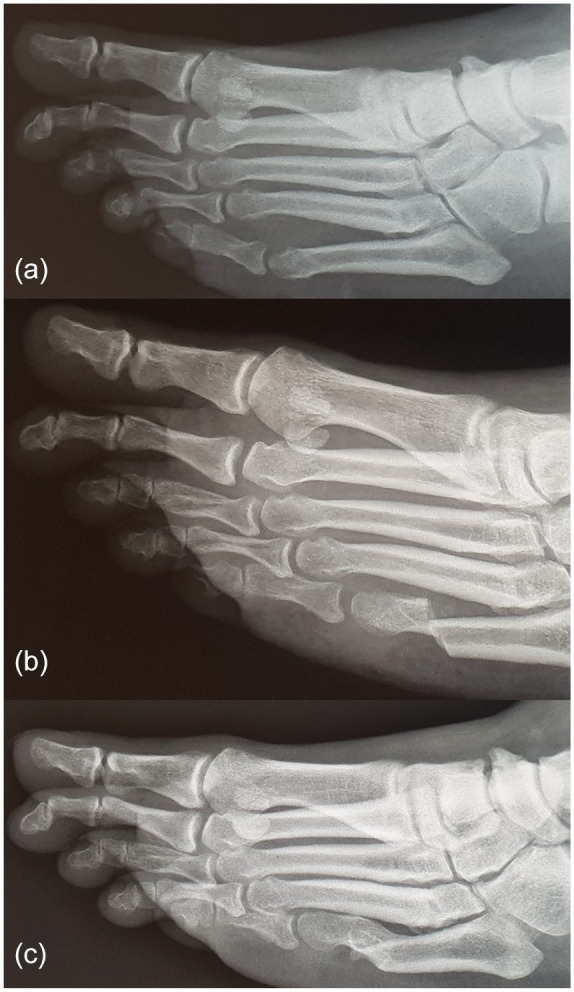
Radiographs of fifth metatarsal: (a) presurgery; (b) 1 week postsurgery; (c) 2 years postsurgery.

The peak pressure under the head of the osteotomized metatarsal decreased from 338.1 to 225.4 kPa (33%, *P* < .0001) following surgery. The pressure time integral under the head of the osteotomized metatarsal decreased from 82.4 kPa·s to 65.0 kPa·s (21%, *P* < .0001). The peak pressures and pressure time integrals broken down by the osteotomized metatarsal are presented in Figure 1. Example clinical photographs and pedobarographs for patient 13 are presented in Figure 2. The peak pressure and pressure time integral decreased under 89% of osteotomized metatarsals and increased in 50% of adjacent metatarsals (Table 2). The pressure under the adjacent metatarsal heads increased in most cases, with the exception of a decrease in pressure under the fifth metatarsal after a fourth metatarsal osteotomy. Radiographic results of a fifth metatarsal osteotomy are presented in Figure 3.

**Table 2.** Metatarsals According to Osteotomy Status and Change in Peak Pressure and Pressure Time Integral.^a^

Relationship to osteotomy	Peak pressure, n (%)			Pressure time integral, n (%)		
	Increased	Same	Decreased	Increased	Same	Decreased
Far	32 (46)	8 (11)	30 (43)	31 (44)	10 (14)	29 (41)
Adjacent	28 (54)	7 (13)	17 (33)	26 (50)	7 (13)	19 (36)
Osteotomized	4 (11)	0	33 (89)	2 (5)	2 (5)	33 (89)

a Change was defined as an increase or decrease of at least ±5%; 32 patients with 5 metatarsals (1 missing data). The peak pressure and pressure time integral decreased under 89% of osteotomized metatarsals and increased in 50% of adjacent metatarsals.

Using multivariate analysis looking at the peak pressures and pressure time integrals after correcting for individual variation and which metatarsal was osteotomized, there was a significant decrease in peak pressure from preoperative (358±16) to postoperative (222±16, *P* < .0001), and in pressure time integrals (147±51 to 79±35 respectively, *P* < .0001).

## Discussion

Operative (or internal) offloading for curing and preventing the recurrence of diabetic foot ulcers is becoming more feasible due to the newer technologies in foot surgery including mini-invasive surgery.^6,30,37,38^ The complication rate with these methods is acceptable. There were fewer complications than the recurrence rate with conservative treatment. But before this treatment can be widely accepted, there is a need to prove the concept that the surgery is indeed achieving its goal of off-loading. Following that, randomized controlled trial–level data will be necessary.^15^ This study is the first to demonstrate that distal metatarsal osteotomy reduces the pressure under a osteotomized metatarsal head. In our patient cohort, distal metatarsal osteotomy reduced both the mean peak pressure and pressure time integral under the osteotomized metatarsal head by 33% and 21%, respectively, both results being clinically and statistically significant. In contrast, there was an increase in plantar pressure under adjacent metatarsals in half of the patient cohort, explaining the development of most of the transfer lesions.

The clinical results we present are similar to those previously reported.^6,30^ Ulcers present for a median of 1.5 months preoperatively were healed at a mean of 3.7 weeks postoperatively (half within 3 weeks and three-fourths within 4 weeks). The operative procedure was relatively safe for these high-risk patients. There were 2 operative site infections (6%) that resolved with antibiotics. Four cases of transfer ulcers and the recurrence under the osteotomy callus were managed successfully, 3 by reoperation and 2 ulcers below the first metatarsal head with shoe modification. There were 4 cases of asymptomatic nonunion of the metatarsal osteotomy. Theoretically, the forefoot transfer lesions may have been minimized by adding a calf-lengthening procedure.^12^ Since we started using metatarsal osteotomies, we have stopped using calf-lengthening procedures, as the hindfoot transfer lesion rates can be as high as 12.9% to 20%,^12^ and heel ulcers are much more difficult to treat and carry a substantial risk of calcaneal osteomyelitis that can lead to a major amputation. Further complications include Achilles tendon rupture and gait disturbances.

Operative off-loading has the advantage of not only curing the ulcer but also in preventing recurrence. The alternative, “conservative treatment" includes total contact casts followed by custom-made orthotics and shoes (the standard for treating neuropathic ulcers below the metatarsal heads). Although effective, this treatment is related to several problems. Casting is a time-consuming expensive procedure and requires skillful personnel. During the treatment, which lasts for several weeks, the patient’s quality of life is considerably compromised, follow-up visits and cast replacement are necessary and there is a considerable complication rate.^17,26^ But the cast only cures the ulcer. Prevention of recurrence requires continued offloading with custom-made orthotics and special shoes. This again requires skillful personnel, is expensive, and requires long-term compliance. Both the expense and the compliance can compromise the success in the population with foot complications of type 2 diabetes mellitus, who are frequently from more socioeconomically deprived background.^2,5^ It is therefore not surprising that the recurrence rate is so high: 40% at 1 year and 60% at 3 years,^4^ which is basically a failure of both the clinical professions and of society to look after these patients. The low recurrence rate we report is very different from that reported by Armstrong et al,^4^ but this comparison may be unfair. Our criteria for recurrence is an ulcer under the same metatarsal. It would seem that most reports relate to any ulcer. Morbach et al clearly separate between a same spot recurrence and new ulcers, but do not state if they included both feet.^25^ Even in the carefully conducted study by Bus et al on the effect of custom-made footwear, it is not clear if they refer to any ulcer in the same foot (probably) or in either foot (possibly).^10^ Molines-Barroso et al differentiate between 41% reulceration and 1% recurrence following metatarsal head resection, the latter more similar to our results.^24^ Jeffcoate et al’s perspective recommends looking at ulcer-free days, a worthy aim our study was not designed for.^19^ Furthermore, if we count any new ulcer and transfer lesion within 1 year as recurrences, our per patient recurrence rate would be 41% (14/34). This difference may result from the different methodology. Our study is based on detailed clinical records on a small patient cohort the senior author was personally acquainted with. In contrast, most other studies have used diagnostic coding data from large databases, which is often limited in its specificity. Each type has its merits and limitations.

This study demonstrated that floating metatarsal osteotomy reduced plantar pressures under the osteotomized metatarsal head. This was coupled with an increase in plantar pressures under the adjacent metatarsal heads, suggesting a transfer of plantar pressures to adjacent areas in the forefoot. These results may help to explain the development of adjacent metatarsal transfer ulcers. Almost all of the heads of adjacent metatarsals demonstrated increased peak pressure and pressure time integral, with the exception of a decrease in pressure under the fifth metatarsal after a fourth metatarsal osteotomy. This may be related to the mobility of the fifth ray, but remains unclear.

We presented data both for peak pressure and pressure time integral. The literature is not clear regarding whether one value could be presented alone. It makes a certain amount of sense that the pressure time integral might be more relevant for ulcer formation.

A clinical limitation of this study is that it evaluated patients with diabetic foot ulcers in the absence of ischemia. Although many DFUs present without ischemia, ischemia complicates things considerably, and treating the ischemia takes priority over most aspects of management aside from debridement of infection or necrotic tissue. Our population may not be representative as the patients’ diabetes was relatively well controlled. Discussing mainly same spot ulcers as mentioned above makes comparing these data to other reports problematic, but the main intention of this study was to present the pressure change. Another possible limitation relates to the data analysis method. The use of the composite-based method for sampling peak pressure could be criticized. It yields slightly higher values than the time-based method. Of course all sampling was done in the same way and analyzed by the same clinician.

In conclusion, this study reinforces the trend for recommending offloading surgery for diabetic foot ulcers with a minimally invasive approach under the metatarsal heads and provides a mechanism for its success.

## Supplemental Material

sj-pdf-1-fai-10.1177_1071100720976099 – Supplemental material for Effect of Mini-invasive Floating Metatarsal Osteotomy on Plantar Pressure in Patients With Diabetic Plantar Metatarsal Head Ulcers Click here for additional data file. Supplemental material, sj-pdf-1-fai-10.1177_1071100720976099 for Effect of Mini-invasive Floating Metatarsal Osteotomy on Plantar Pressure in Patients With Diabetic Plantar Metatarsal Head Ulcers by Eran Tamir, Michael Tamar, Moshe Ayalon, Shlomit Koren, Noam Shohat and Aharon S. Finestone in Foot & Ankle International

V2_etc._research_data_Withdrawn – Research Data for Effect of Mini-invasive Floating Metatarsal Osteotomy on Plantar Pressure in Patients With Diabetic Plantar Metatarsal Head Ulcers Click here for additional data file. Research Data, V2_etc._research_data_Withdrawn for Effect of Mini-invasive Floating Metatarsal Osteotomy on Plantar Pressure in Patients With Diabetic Plantar Metatarsal Head Ulcers by Eran Tamir, Michael Tamar, Moshe Ayalon, Shlomit Koren, Noam Shohat and Aharon S. Finestone in Foot & Ankle International https://creativecommons.org/licenses/by/4.0/ This article is distributed under the terms of the Creative Commons Attribution 4.0 License ( https://creativecommons.org/licenses/by/4.0/) which permits any use, reproduction and distribution of the work without further permission provided the original work is attributed as specified on the SAGE and Open Access pages ( https://us.sagepub.com/en-us/nam/open-access-at-sage).
