# Supplementary material for: Effect of Mini-invasive Floating Metatarsal Osteotomy on Plantar Pressure in Patients With Diabetic Plantar Metatarsal Head Ulcers
Source: Foot Ankle Int. 2020 Dec 17;42(5):536–43. doi: 10.1177/1071100720976099 (PMC8127671; doi:10.1177/1071100720976099)
Supplement: V2_etc._research_data_Withdrawn – Research Data for Effect of Mini-invasive Floating Metatarsal Osteotomy on Plantar Pressure in Patients With Diabetic Plantar Metatarsal Head Ulcers [file V2_etc._research_data_Withdrawn.pdf]

Retraction of Supplemental Material for 'Effect of Mini-invasive Floating Metatarsal Osteotomy on Plantar Pressure in Patients With Diabetic'

The Research Data supplemental material associated with Tamir E, Tamar M, Ayalon M, Koren S, Shohat N, Finestone AS. Effect of Mini-invasive Floating Metatarsal Osteotomy on Plantar Pressure in Patients With Diabetic Plantar Metatarsal Head Ulcers. *Foot & Ankle International*. December 2020. doi:10.1177/1071100720976099, has been removed for the following reason(s): accidental inclusion of identifying information was made. To protect patient privacy, the Editors have removed the Research Data file from Online First.

If you have a concern about this, please contact SAGE.
